# Strategic Use of Vegetable Oil for Mass Production of 5-Hydroxyvalerate-Containing Polyhydroxyalkanoate from δ-Valerolactone by Engineered *Cupriavidus necator*

**DOI:** 10.3390/polym16192773

**Published:** 2024-09-30

**Authors:** Suk-Jin Oh, Yuni Shin, Jinok Oh, Suwon Kim, Yeda Lee, Suhye Choi, Gaeun Lim, Jeong-Chan Joo, Jong-Min Jeon, Jeong-Jun Yoon, Shashi Kant Bhatia, Jungoh Ahn, Hee-Taek Kim, Yung-Hun Yang

**Affiliations:** 1 Department of Biological Engineering, College of Engineering, Konkuk University, Seoul 05029, Republic of Korea; equal73@naver.com (S.-J.O.); sdbsdl0526@naver.com (Y.S.); xmfvm@naver.com (J.O.); rlatn990@naver.com (S.K.); karecurry@konkuk.ac.kr (Y.L.); suhye0823@konkuk.ac.kr (S.C.); lge0919@naver.com (G.L.); shashikonkukuni@konkuk.ac.kr (S.K.B.); 2 Department of Chemical Engineering, Kyung Hee University, Yongin-si 17104, Gyeonggi-do, Republic of Korea; jcjoo@khu.ac.kr; 3 Department of Green & Sustainable Materials R&D, Research Institute of Clean Manufacturing System, Korea Institute of Industrial Technology (KITECH), Cheonan-si 31056, Chungcheongnam-do, Republic of Korea; j2pco@kitech.re.kr (J.-M.J.); jjyoon@kitech.re.kr (J.-J.Y.); 4 Institute for Ubiquitous Information Technology and Application, Konkuk University, Seoul 05029, Republic of Korea; 5 Biotechnology Process Engineering Center, Korea Research Institute Bioscience Biotechnology (KRIBB), Cheongju-si 28116, Chungcheongbuk-do, Republic of Korea; ahnjo@kribb.re.kr; 6 Department of Food Science and Technology, Chungnam National University, Daejeon 34134, Republic of Korea

**Keywords:** polyhydroxyalkanoates, 5-hydroxyvalerate, δ-valerolactone, plant oil

## Abstract

Although efforts have been undertaken to produce polyhydroxyalkanoates (PHA) with various monomers, the low yield of PHAs because of complex metabolic pathways and inhibitory substrates remains a major hurdle in their analyses and applications. Therefore, we investigated the feasibility of mass production of PHAs containing 5-hydroxyvalerate (5HV) using δ-valerolactone (DVL) without any pretreatment along with the addition of plant oil to achieve enough biomass. We identified that PhaC_BP-M-CPF4_, a PHA synthase, was capable of incorporating 5HV monomers and that *C. necator* PHB^−4^ harboring *phaC*_BP-M-CPF4_ synthesized poly(3HB-*co*-3HHx-*co*-5HV) in the presence of bean oil and DVL. In fed-batch fermentation, the supply of bean oil resulted in the synthesis of 49 g/L of poly(3HB-*co*-3.7 mol% 3HHx-*co*-5.3 mol%5HV) from 66 g/L of biomass. Thermophysical studies showed that 3HHx was effective in increasing the elongation, whereas 5HV was effective in decreasing the melting point. The contact angles of poly(3HB-*co*-3HHx-*co*-5HV) and poly(3HB-*co*-3HHx) were 109 and 98°, respectively. In addition, the analysis of microbial degradation confirmed that poly(3HB-*co*-3HHx-*co*-5HV) degraded more slowly (82% over 7 days) compared to poly(3HB-*co*-3HHx) (100% over 5 days). Overall, the oil-based fermentation strategy helped produce more PHA, and the mass production of novel PHAs could provide more opportunities to study polymer properties.

## 1. Introduction

Polyhydroxyalkanoates (PHAs) are polymers that accumulate within microorganisms in nutrient-limited environments, and they have been studied as promising alternatives to conventional petroleum-based plastics because of their biodegradability and bio-based characteristics [1]. Moreover, the excellent biodegradability of PHAs, which decompose not only in soil but also in marine environments, makes them attractive candidates for use as bio-plastics [2]. PHAs are composed of various hydroxyalkanoate monomers that are present in microorganisms [3]. The most common PHA is poly(3-hydroxybutyrate) (P(3HB)), which consists of a 3HB monomer. P(3HB) had the advantage of being naturally produced in various microorganisms without the need for engineering [4]. However, its rigid and brittle nature, along with its high melting point, makes industrial utilization of PHB difficult [5]. These issues have been addressed by integrating other monomers into the polymer chains to synthesize copolymers, terpolymers, and tetrapolymers [6].

Poly(3-hydroxybutyrate-*co*-5-hydroxyvalerate) (PHB5HV or P(3HB-*co*-5HV)) is an actively researched PHA copolymer. 5-hydroxyvalerate (5HV) contributes to the flexibility of PHA polymers and decreases their melting points [7]. Additionally, the incorporation of 5HV into a polymer enhances its degradation rate by lipases; P(3HB-*co*-3HP-*co*-5HV) has been reported to exhibit low cytotoxicity and support cell proliferation [8]. Poly(3HB-*co*-5HV) can be synthesized by *Aneurinibacillus thermoaerophilus* and *Methylocystis parvus* [9,10]. One of the most studied PHA-producing strains, wild-type *Cupriavidus necator*, was also reported to be able to synthesize PHA containing 5HV; however, the mole fraction of 5HV in PHA produced by *C. necator* was very low [11]. Nonetheless, PHB5HV with a high mole fraction of 5HV could be synthesized by simply introducing PHA synthase with broad specificity into *C. necator*. Recently, in our lab, we successfully produced poly(3HB-*co*-5HV) with the mole fraction of 5HV reaching 70% using an engineered strain of *C. necator*. The PHB5HV produced by this strain had an increased elongation at a break of up to 1400% [12]. However, despite the favorable properties of PHAs containing 5HV monomers, their production is still relatively low, and further research on large-scale production is needed.

*C. necator*, also known as *Ralstonia eutropha*, can utilize various carbon sources, such as fructose, CO_2_, and plant oils, to produce PHA [13]. To date, various studies have been conducted to synthesize different types of PHA from fructose by constructing pathways for monomer synthesis through genetic engineering in *Cupriavidus necator* or by supplying precursors alongside fructose [14,15,16,17]. However, producing various PHAs from fructose through genetic modification significantly reduces the yield of PHA, and in subsequent scale-up processes, it may not be possible to produce PHA with the desired mole fraction of monomers [18,19] (Table 1). Moreover, considering that the highest reported PHB yield from fructose fermentation is 18.46 g/L from 350 g/L of fructose, the method of supplying precursors along with fructose is also not suitable for the mass production of various PHAs.

Plant oils, such as soybean oil, palm oil, rapeseed oil, and jatropha oil, have been widely used as inexpensive carbon sources for PHA production in *C. necator*, aiming to reduce the cost of PHA [25]. Furthermore, plant oils yield higher amounts of PHA than sugars, and with simple genetic modifications to *C. necator*, plant oils can be used to produce poly (3-hydroxybutyrate-*co*-3-hydroxyhexanoate) (PHBHHx or poly(3HB-*co*-3HHx)) with properties superior to those of PHB [26,27]. Engineered *C. necator* H16 produced 125.9 g/L of PHBHHx from 164.7 g/L of dry cell weight using palm kernel oil. Another study reported the production of 86% PHBHHx from 124 g/L biomass of *C. necator* Re2058/pCB113 using fructose and rapeseed oil [28,29]. Therefore, using plant oil instead of fructose for the synthesis of various PHAs in *C. necator* seems more advantageous in terms of yield and cost-effectiveness.

While plant oils have been widely utilized to produce poly(3HB-*co*-3HHx), research on their use for producing PHAs with other monomers remains limited. Therefore, this study aims to assess the feasibility of using vegetable oil and precursors to enable large-scale production of various PHAs, particularly focusing on 5HV monomers by co-supplying DVL with vegetable oil. Furthermore, by analyzing the thermal and physical properties of the resulting polymer, this research provides a deeper understanding of the role of 5HV within PHA.

## 2. Materials and Methods

### 2.1. Microorganisms and Culture Conditions

**Figure 1:**
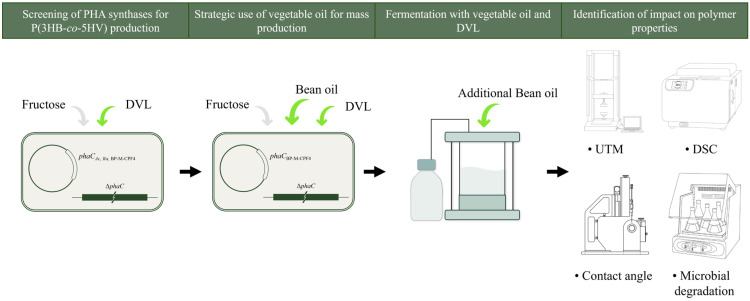
The overall methodological framework of the experiment. The experiment proceeded in the following order: establishing a strain capable of producing poly(3HB-*co*-5HV), confirming the increase in PHA production containing 5HV through vegetable oil feeding, verifying the feasibility of mass-producing PHA containing 5HV through vegetable oil-based fermentation, and finally, analyzing the physical properties of the produced PHA.

The *C. necator* PHB^−4^ wild-type strain and PHB^−4^ strains harboring various PHA synthase genes (*phaC*_BP-M-CPF4_, *phaC_Ac_*, and *phaC_Ra_*) were cultured in 5 mL of tryptic soy broth (TSB) at 30 °C for 24 h to obtain the seed culture (Figure 1).

The culture was supplemented with 50 µg/mL kanamycin for plasmid activity. For PHA production, 5× *Ralstonia eutropha* minimal medium (5× ReMM; 20 g/L NaH_2_PO_4_, 23 g/L Na_2_HPO_4_, 2.25 g/L K_2_SO_4_), 100× MgSO_4_ (39 g/L MgSO_4_), 100× CaCl_2_ (6.2 g/L CaCl_2_), and 1000× trace element (15 g/L FeSO_4_·7H_2_O, 2.4 g/L MnSO_4_·H_2_O, 2.4 g/L ZnSO_4_·7H_2_O, 0.48 g/L CuSO_4_·5H_2_O dissolved in 0.1 M hydrochloric acid) solutions were used. The 5× ReMM was sterilized at 121 °C for 15 min using an autoclave, and the 100× MgSO_4_, 100× CaCl_2_, and 1000× trace element solutions were filtered using a 28-mm syringe filter with a 0.22-μm polyethersulfone (PES) membrane (Sartorious, Goettingen, Germany). A fructose solution (200 g/L) was used as the carbon source, and 50 g/L of urea solution was used as the nitrogen source. The production test of PHA with 3HHx and 5HV units was carried out in a 5 mL culture in a 14 mL round test tube, and the main culture conditions were as follows: 4 g/L NaH_2_PO_4_, 4.6 g/L Na_2_HPO_4_, 0.45 g/L K_2_SO_4_; 0.39 g/L MgSO_4_; 0.062 g/L CaCl_2_; 0.0015 g/L FeSO_4_·7H_2_O, 0.0024 g/L MnSO_4_·H_2_O, 0.0024 g/L ZnSO_4_·7H_2_O, 0.00048 g/L CuSO_4_·5H_2_O; 10 g/L fructose; 1 g/L urea. Additionally, various concentrations of 5-hydroxyvaleric acid (5HVA), δ-valerolactone (DVL), and bean oil were added, and 50 µg/mL kanamycin was added for plasmid activity. Unless otherwise specified, medium components were purchased from Sigma-Aldrich (St. Louis, MO, USA).

### 2.2. Analytical Methods

For PHA analysis, the culture broth was centrifuged, and the resulting pellet was washed twice with 1 mL distilled water (DW) and 1 mL hexane. Hexane was used to remove the residual bean oil and was not used when bean oil was not added to the culture. The cell pellet was washed once again with 1 mL of distilled water. The washed cells were then transferred into a glass vial for lyophilization, and the dry cell weight was measured. Next, 1 mL of chloroform and 1 mL of 15% (*v*/*v*) H_2_SO_4_/85% methanol solution were added to a glass vial, and methanolysis was performed at 100 °C for 2 h, followed by cooling to room temperature. Then, 1 mL of DW was added to the methyl ester solution, and the mixture was vortexed twice for 5 s each. The bottom of the chloroform layer was transferred to a microtube containing anhydrous Na_2_SO_4_ to remove residual water. The filtered 1 mL sample was analyzed using GC-FID (Young In Chromass 6500, Seoul, Republic of Korea) equipped with a fused silica capillary column (DB-FFAP, 30-mm length, 0.320-mm internal diameter, and 0.25 film, Agilent, Santa Clara, CA, USA). The injection volume was 1 µL, and the split ratio was 1/10. Helium was used as the carrier gas at a flow rate of 3.0 mL/min. The oven program for PHA analysis was as follows: 80 °C for 5 min, then increased from 80 °C to 220 °C at a rate of 20 °C/min, and held at 220 °C for 5 min. During the analysis, the injector temperature was maintained at 210 °C, and the FID temperature was maintained at 230 °C.

### 2.3. Gel Permeation Chromatography (GPC)

GPC was used to determine the number-average molar mass (Mn), weight-average molar mass (MW), and dispersity. GPC was performed using an HPLC system (Young In Chromass, Seoul, Republic of Korea) comprising a loop injector (Rheodyne 7725i), dual-headed isocratic pump system (YL9112), column oven (YL9131) with three columns (K-G 4A, guard column; K-804 8.0 × I.D. × 300 mm; K-805, 8.0 × 300 mm; Sho-dex), and a refractive index detector (YL9170). Chloroform was used as the mobile phase at a flow rate of 1 mL/min at 40 °C. The injection volume of the prepared samples was 20 µL. Polystyrene standards ranging from 5000 to 2,000,000 Da were used to calculate the MW and construct the calibration curve.

### 2.4. Analysis of the Physical and Thermal Characteristics

A Universal Testing Machine (UTM) Model was used to measure the tensile strength, Young’s modulus, and elongation at break of the samples. The samples were cut into 10 × 60-mm pieces, and the gauge length was defined accordingly. The tests were conducted at a crosshead speed of 10 mm/min. The elongation at break was calculated using Equation (1):

*EL* = (*d_after_* − *d_before_*)/*d_before_* × 100 (1)

where *d* represents the distance between the grips holding the sample before and after the sample break.

Differential scanning calorimetry (DSC) was performed using a NEXTA DSC 200 instrument (Hitachi high-tech, Hitachi, Japan) to analyze the thermal properties of the PHAs. Approximately 5 mg of the PHA film containing 3HHx and 5HV units was measured in an aluminum pan for DSC. The experiment was conducted under a N_2_ atmosphere. The heating and cooling rates were all 10 °C/min. The temperature program is as follows: 30 °C, 7 min → −60 °C, 10 min → 190 °C, 10 min (first heating) → −60 °C, 2 min → 30 °C, 10 min → −60 °C, 10 min → 190 °C, 10 min (second heating) → −60 °C, 0 min. The crystallization temperature (Tc), glass transition temperature (Tg), and melting temperature (Tm) of the polymer were determined via second heating.

### 2.5. Culture Conditions for a 5-L Fermenter

Precultures for the fermenter were prepared at a volume of 50 mL (TSB) in two 250-mL baffled flasks at 30 °C for 24 h. Each culture was centrifuged at 3511× *g* at 4 °C. The cell pellet was washed twice with 10 mL of DW. Each cell pellet was then suspended in 10 mL of DW to inoculate the fermenter with a total of 20 mL of cell culture. Fed-batch fermentation was conducted on a 2-L scale in a 5-L fermenter. The main culture conditions at the beginning of the culture were as follows: 4 g/L NaH_2_PO_4_, 4.6 g/L Na_2_HPO_4_, 0.45 g/L K_2_SO_4_; 0.39 g/L MgSO_4_; 0.062 g/L CaCl_2_; 0.0015 g/L FeSO_4_·7H_2_O, 0.0024 g/L MnSO_4_·H_2_O, 0.0024 g/L ZnSO_4_·7H_2_O, 0.00048 g/L CuSO_4_·5H_2_O; 10 g/L fructose; 5 g/L Bean oil; 1 g/L NH_4_NO_3_. Additionally, 100 g/L of bean oil was gradually supplied for 10–20 h, which is the exponential phase, and 5 g/L of DVL was supplied at 48 h in one stroke. For pH control, phosphoric acid was used as the acid, and ammonia water as the base. The pH was adjusted to 6.8, and the dissolved oxygen (DO) level was set at 20%. The initial stirring rate was 200 rpm and was increased to 600 rpm to maintain the DO level during culture.

## 3. Results

### 3.1. PhaC Screening for Efficient 5HV Polymerization in PHA

Wild-type *C. necator* has been reported to synthesize PHB5HV [11]. However, because of the high specificity of *C. necator phaC* for 3HB, the mole fraction of 5HV was very low. Additionally, during PHA mass production, if a large amount of carbon source was supplied to produce 3HB monomers, it was expected that the mole fraction of 5HV would decrease further because of the accumulation of large amounts of 3HB in PHA. Therefore, for the efficient production of PHAs containing 5HV, screening for *phaC* with broad specificity not only toward 3HB but also toward 5HV was necessary. In addition, as 5HV is obtained by an additional saponification process requiring an increase in pH using DVL for ring opening and then neutralization of the pH for biological utilization, the ability of strains to use DVL as a precursor was also important [12,30].

To confirm the individual activity of *phaC*, a plasmid carrying three types of *phaC* (*phaC*_BP-M-CPF4_, *phaC_Ac_*, and *phaC_At_*) was inserted into *C. necator* PHB^−4^. *C. necator* PHB^−4^ is a strain that cannot produce PHB because of the abnormal expression of PHA synthase from *C. necator* H16, achieved through random mutagenesis techniques [31,32]. Next, we tested the ability of the constructed strains to synthesize poly (3HB-*co*-5HV) using delta-valerolactone as a precursor; along with wild-type *C. necator* H16; 1% fructose was used to support cell growth.

**Figure 2:**
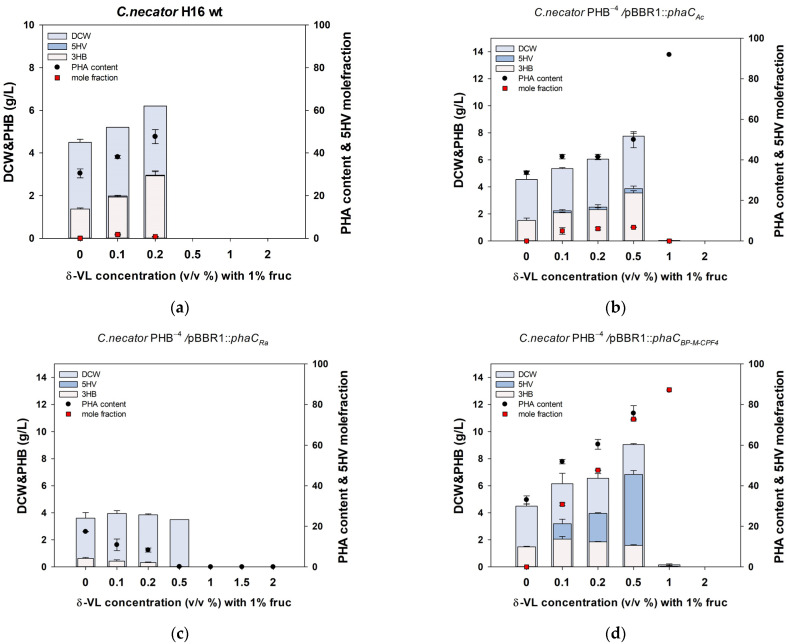
Validation of the 5HV polymerization ability of wild-type *Cupriavidus necator* H16 and *C. necator* PHB^−4^ harboring various PHA synthases. (**a**) Wild-type *C. necator* H16 (**b**) *C. necator* PHB^−4^ harboring *phaC_Ac_* (**c**) *C. necator* PHB^−4^ harboring *phaC_Ra_* (**d**) *C. necator* PHB^−4^ harboring *phaC*_BP-M-CPF4_. Statistical analysis was performed by applying ANOVA with the level of significance at 5%.

Wild-type *C. necator* H16 was able to synthesize poly (3HB-*co*-5HV) from DVL. The highest achieved mole fraction of 5HV in PHA was 1.7%, which was noted at a DVL concentration of 0.1%; however, it decreased to 0.7% when the DVL concentration was 0.2% (Figure 2a). As the DVL concentration increased, both dry cell weight (DCW) and PHB also increased. This indicates that DVL contributes to 3HB accumulation in *C. necator*. However, when the concentration of DVL exceeded 0.5%, *C. necator* H16 wild type did not grow.

*phaC_Ac_* and *phaC_Ra_* from *Aeromonas caviae* and *Rhodococcus aetherivorans*, respectively, have been widely used to synthesize poly(3HB-*co*-3HHx) in *C. necator* because of their broad specificity [33,34,35,36]. *phaC_Ac_* and *phaC_Ra_* were codon-optimized and inserted into the pBBR1MCS2 vector along with a high-expression ribosome binding site (RBS). As expected, *C. necator* PHB^−4^ with inserted *phaC_Ac_* produced PHA with a 5HV mole fraction of approximately 6% from DVL, with the highest 5HV mole fraction of 6.7% being achieved at a DVL concentration of 0.5% (Figure 2b). However, *C. necator* PHB^−4^ carrying *phaC_Ra_* was unable to synthesize poly(3HB-*co*-5HV). Additionally, as the concentration of 5HV increased, the PHA content decreased (Figure 2c).

*phaC*_BP-M-CPF4_, discovered by a research team in Malaysia, is a PHA synthase derived from uncultured bacteria found through the metagenomic analysis of mangrove soil. *phaC*_BP-M-CPF4_ has been reported to possess broad substrate specificity and is capable of polymerizing 3HB, but also 3HV, 4HB, 4HV, 5HV, 3HHx, and others [37,38,39]. Furthermore, when codon-optimized *phaC*_BP-M-CPF4_ is expressed in *C. necator* PHB^−4^ with a high-expression RBS, it can synthesize poly(3HB-*co*-5HV) with a mole fraction of 70% 5HV from 5-hydroxyvaleric acid (5-HVA) [12]. The PHA synthase with the highest 5HV polymerization ability was *phaC*_BP-M-CPF4_. When we confirmed the production of poly (3HB-*co*-5HV) from DVL in *C. necator* PHB^−4^ with inserted *phaC*_BP-M-CPF4_ (PHB^−4^/BP), we observed that with increasing DVL concentration, both the production of PHA and the mole fraction of 5HV in PHA increased (Figure 2d). PHB^−4^/BP produced poly(3HB-*co*-5HV) with a 5HV mole fraction of 72.7% at a concentration of 0.5% DVL, yielding 6.85 ± 0.20 g/L. This is comparable to a previous study in which the same strain produced PHA with a 5HV mole fraction of 72.2%, yielding 5.87 ± 0.10 g/L when 0.5% 5HVA was used as the precursor [12]. The similarity in the 5HV mole fraction and PHA production when DVL and its ring-opening form, 5HVA, were used suggests that *C. necator* PHB^−4^ expresses a lactonase capable of opening lactone rings, such as DVL, and its activity was already sufficient.

### 3.2. Validation of Plant Oil for Increased Production of PHA Containing 5HV

Plant oil has been widely used to enhance the price competitiveness of PHA because of its cost-effectiveness and high conversion rate to PHA compared to sugar [40,41]. In addition, plant oils could help in the production of considerable amounts of cell mass and PHA [21,29,42,43,44]. Therefore, we aimed to investigate whether additional supplementation with plant oil could increase the production of poly(3HB-*co*-5HV) by the PHB^−4^/BP strain to maximize the production of PHA containing 5HV. Additionally, in a previous study, considering the replacement of the native-*phaC* of *C. necator* H16 with the broad-specificity *phaC* from *Rhodococcus aetheriborans*, resulting in the production of PHA with a 1–1.5% mole fraction of 3HHx, we anticipated that feeding the PHB^−4^/BP strain with an oil source would lead to the integration of additional 3HHx into PHA, thereby enhancing the flexibility of polymer [45].

**Figure 3:**
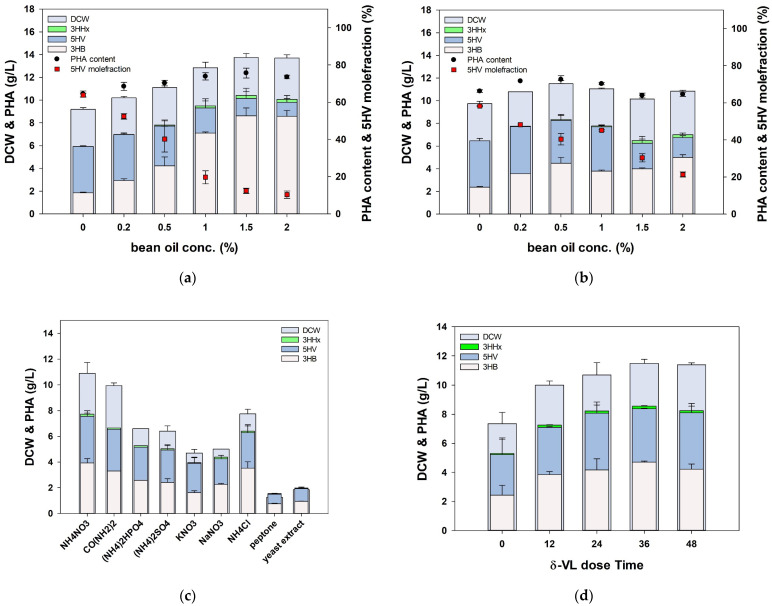
Confirmation of increased production of poly(3HB-*co*-3HHx-*co*-5HV) after supplying bean oil. (**a**) 5-hydroxyvaleric acid (5HVA) or (**b**) δ-valerolactone (DVL) was used as the 5HV precursor. (**c**) N-source optimization for poly(3HB-*co*-3HHx-*co*-5HV) production. (**d**) DVL feeding time optimization. Statistical analysis was performed by applying ANOVA with a level of significance at 5%.

In the previous experiment, we confirmed that the optimal concentration of DVL to produce poly(3HB-*co*-5HV) with a high 5HV mole fraction was 0.5% (Figure 2). Therefore, we used 1% fructose as the carbon source and 0.5% 5HV or DVL as the precursors, with bean oil supplemented at concentrations ranging from 0% to 2%. We observed that with the use of 5HV as the precursor, the addition of bean oil from 0% to 1.5% led to an increase in both PHA production and DCW (Figure 3a). Additionally, as expected, a small amount of 3HHx was polymerized, resulting in the formation of a poly(3HB-*co*-3HHx-*co*-5HV) ter-polymer. However, as the concentration of bean oil increased beyond 1.5%, the mole fraction of 5HV in PHA decreased from 64% to 10%.

On the contrary, when DVL was used as the precursor, increasing the concentration of bean oil from 0% to 0.5% resulted in an approximately 30% increase in PHA production (Figure 3b). However, when bean oil was added at concentrations of 1% or higher, both DCW and PHA decreased. This decrease might be attributed to the fact that 5HVA, as a free fatty acid, could directly participate in beta-oxidation along with bean oil, whereas DVL, as a lactone, could not directly participate in beta-oxidation. Therefore, the reduced compatibility between bean oil and DVL may be attributed to the requirement for lactonase, an enzyme essential for the ring-opening process of DVL. However, 5HVA is generally difficult to obtain, and an additional saponification process is required. Therefore, we focused on the 30% increase in PHA production when bean oil was added to the DVL precursor and designed further experiments.

The type of nitrogen source is crucial for PHA production from various substrates [46]. Furthermore, since the carbon source has changed, the corresponding control of the nitrogen source is also necessary. To enhance PHA production from bean oil and DVL, after including 1% fructose for initial cell growth, nine different nitrogen sources were administered at a concentration of 0.1%, and the production of poly(3HB-*co*-3HHx-*co*-5HV) was compared (Figure 3c). When urea and NH_4_Cl were employed, the PHA production was similar, yielding 6.66 g/L and 6.41 ± 0.35 g/L, respectively. However, the PHA content when urea was used was 67.9%, whereas it was higher at 82.6 ± 1.85% when NH_4_Cl was used. On the contrary, when NH_4_NO_3_ was used, PHB^−4^/BP accumulated the highest PHA at 7.73 ± 0.19 g/L, and the DCW was also the highest at 10.9 ± 0.6 g/L. In our study, the type of nitrogen source used resulted in significant differences in PHA production from bean oil and DVL. Notably, the highest cell mass and PHA yield were achieved when using NH_4_NO_3_, a less commonly used nitrogen source, instead of the more commonly used sources such as urea and (NH_4_)_2_SO_4_ in *Cupriavidus necator* fermentation. This highlights the importance of optimizing the nitrogen source as a key aspect of fermentation optimization.

The precursors used in the synthesis of PHA copolymers are known to inhibit cell growth [47]. Previous experiments revealed that DVL, the precursor of 5HV, also inhibited cell growth at concentrations of 0.5–1%. The toxicity of such precursors could be mitigated by supplying precursors after the cells had grown to a certain extent following the initiation of the culture. To confirm that the delayed supply of the DVL precursor could overcome its toxicity, DVL was supplied at 0, 12, 24, 36, and 48 h after the start of the culture, and PHA production was subsequently observed. It was observed that the delayed supply of DVL resulted in higher PHA production, with the production increasing with the extent of delay. When DVL was supplied at 0 h, the DCW and PHA production were 7.35 ± 0.55 g/L and 5.31 ± 0.75 g/L, respectively (Figure 3d). When DVL supply was delayed, both DCW and PHA production increased, reaching a maximum of 11.5 ± 0.2 g/L DCW and 8.56 ± 0.02 PHA, respectively.

### 3.3. Fed-Batch Production of Poly(3HB-co-3HHx-co-5HV)

Based on the previously identified conditions, the feasibility of mass-producing poly(3HB-*co*-3HHx-*co*-5HV) using PHB^−4^/BP was tested on a 5-L scale. The working volume in the 5-L fermenter was 2 L. Initially, 1% fructose, 0.5% bean oil, and 0.1% NH_4_NO_3_ were fed during cultivation, with 0.5% DVL added after 48 h of fermentation, and terpolymer production and cell growth of the PHB^−4^/BP strain were observed. At 42 h, i.e., 6 h before DVL feeding, a PHA production of 0.57 ± 0.01 g/L was observed, with a PHA content of 9.18 ± 0.34%. The low PHA production and PHA content in the fermenter were attributed to the use of ammonia water as a pH regulator. Owing to the continuous decrease in pH with the growth of PHB^−4^/BP, ammonia water was continuously supplied to maintain the pH, leading to excessive N-source feeding and a subsequent decrease in PHA content. However, after DVL feeding at 48 h, the 5HV molar fraction continued to increase until 84 h, resulting in 3.17 ± 0.06 g/L of poly(3HB-*co*-2 mol% 3HHx-*co*-76 mol% 5HV) at 84 h.

**Figure 4:**
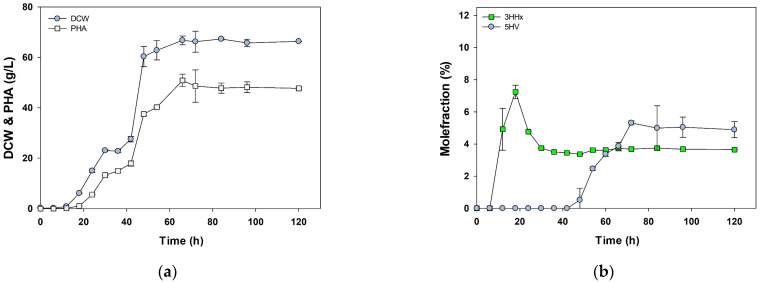
Fed-batch Poly(3HB-*co*-3HHx-*co*-5HV) production by *C. necator* PHB^−^^4^ harboring *phaC*_BP-M-CPF4_ in a 5-L jar fermenter. The initial culture conditions included 1% fructose, 0.5% bean oil, and 0.1% NH_4_NO_3_. From 10 h to 20 h of culture, 100 g/L of bean oil was supplied, and 5 g/L of DVL was added after 48 h. The changes in (**a**) DCW (Dry Cell Weight) and PHA, as well as (**b**) the molar fractions of 3HHx and 5HV, were monitored over the cultivation period. Statistical analysis was performed by applying ANOVA with the level of significance at 5%.

To increase PHA production and content, an additional 100 g/L of bean oil was supplied from 10 h to 20 h with all other conditions remaining unchanged. Consequently, both DCW and PHA increased until 72 h, accumulating 48.63 ± 4.53 g/L of poly(3HB-*co*-3.7 mol% 3HHx-*co*-5.3 mol% 5HV) with 66.25 ± 2.95 g/L of DCW (Figure 4). Subsequently, when the supply of bean oil was increased to 200 g/L from 10 h to 20 h, 68.6 ± 7.8 g/L of poly(3HB-*co*- 5.6 mol% 3HHx-*co*- 2.7 mol% 5HV) was accumulated at 144 h with 90.3 ± 0.9 g/L of DCW (Supplementary Figure S1). However, the growth rate decreased, possibly because of the reduced oxygen supply resulting from the rapid supply of oil over a short period. Through this experiment, we confirmed the potential for the mass production of PHA-containing 5HV monomers using the PHB^−4^/BP strain with bean oil supplementation.

### 3.4. Physical and Mechanical Properties of Poly(3HB-co-3HHx-co-5HV)

To characterize the produced Poly (3HB-*co*-3.6 mol% 3HHx-*co*-4.9 mol% 5HV), the cell pellet was concentrated using a continuous centrifuge. PHA was then extracted using chloroform and cast into films. The thermal and physical properties of the poly(3HB-*co*-3HHx-*co*-5HV) film were analyzed using a UTM, DSC, and GPC and compared with those of the poly(3HB-*co*-7.1 mol% 3HHx) film.

In a previous study, a poly(3HB-*co*-5HV) film with a 70% 5HV mole fraction was reported to exhibit an elongation at a break of 1400% [12]. However, the increase in elongation at break owing to the 5HV content in PHA was found to be minimal at low 5HV mole fractions. Upon confirming the elongation at break using UTM for both films, the elongation at break of poly(3HB-*co*-7.1 mol% 3HHx) was 176.6 ± 18.2%, whereas that of Poly(3HB-*co*- 3.6 mol% 3HHx-*co*- 4.9 mol% 5HV) was 16.6 ± 1.4% (Table 2). This indicates that at low mole fractions, 3HHx has a more significant effect on elongation at break than 5HV.

The high melting point of PHB hinders its industrial application because it requires more energy for plastic molding. Additionally, the degradation of PHA at high temperatures is unavoidable, which can lead to changes in the polymer’s properties. Therefore, to facilitate the molding of PHA to increase its industrial applicability and prevent degradation during the molding process, it is necessary to decrease its melting point. Analysis of the thermal behavior of the two films using DSC revealed that the Tm of Poly(3HB-*co*-3HHx-*co*-5HV) was 151.5 °C, and no Tg and Tc were observed (Table 3). In contrast, for poly(3HB-*co*-3HHx), the Tm was 175.5 °C, with Tg and Tc values of 2.5 °C and 48.3 °C, respectively. Considering the significantly lower Tm of Poly(3HB-*co*-3HHx-*co*-5HV) than that of poly(3HB-*co*-3HHx), it can be inferred that the 5HV monomer in PHA contributes more to the decrease in Tm than 3HHx.

The roles of 3HHx and 5HV as PHA monomers have also been confirmed in two previous studies that produced poly(3HB-*co*-33 mol% 5HV) and poly(3HB-*co*-31.1 mol% 3HHx) using engineered *C. necator* [12,22]. The elongation at break for poly(3HB-*co*-31.1 mol% 3HHx) produced from lauric acid was 243.4 ± 36.4%, whereas poly(3HB-*co*-33 mol% 5HV) exhibited an elongation at break of 157.7%. Furthermore, PHBHHx achieved an elongation at a break of 132.2 ± 17.3%, similar to that of poly(3HB-*co*-33 mol% 5HV), with just the incorporation of 9.4% of 3HHx. Regarding thermal properties, poly(3HB-*co*-33 mol% 5HV) exhibited melting points of 31.5 °C and 153.3 °C, whereas poly(3HB-*co*-31.1 mol% 3HHx) showed a relatively higher melting point of 166.4 °C. A relatively lower melting point was observed in the presence of 5HV, even at low mole fractions. Poly(3HB-*co*-12.0 mol% 3HHx) produced from *Cupriavidus eutrophus* B10646 had a melting point of 170 °C, whereas poly(3HB-*co*-5HV) produced from *Escherichia coli* showed a melting point of 159.2 °C with only 4.7 mol% polymerization of 5HV [48,49].

For commercialization and improved processability of PHA, enhancement of flexibility and reduction of melting point should be achieved simultaneously. Typically, increasing the content of monomers other than 3HB in PHA copolymers improves the flexibility of the polymer and reduces its melting point. However, the mole fraction of the monomers required to achieve ideal mechanical properties and melting points may vary. For instance, in PHBHHx, a mole fraction of approximately 10% 3HHx may be optimal because higher 3HHx mole fractions result in amorphous polymers with reduced tensile strength and Young’s modulus. However, a higher molar fraction of 3HHx may be required to achieve a lower melting point. In such cases, the production of PHA terpolymers by incorporating additional monomers with different properties is a good solution.

This study confirmed that 3HHx is effective in increasing elongation even at low molar fractions, whereas 5HV is effective in reducing the melting point at low molar fractions. Therefore, it is anticipated that optimizing the molar fraction of 3HHx to achieve the desired mechanical properties, followed by the addition of 5HV to achieve a lower melting point, can be an effective approach.

### 3.5. Comparison of PHBHHx5HV and Conventional PHBHHx Degradation and Contact Angle

Previous reports have identified the role of 5HV units in facilitating polymer degradation of PHA by pig pancreatic lipase [50]. However, the role of 5HV in PHA degradation by microorganisms has not been reported. *Microbulbifer* sp. Sol66, a PHB-degrading strain isolated from the coastal area of Korea, demonstrated a rapid degradation rate, reaching up to 98% in just 4 days of cultivation [51]. To verify the role of 5HV in PHA degradation by microorganisms, the degradation rate of Poly(3HB-*co*-3.6 mol% 3HHx-*co*-4.9 mol% 5HV) by Sol66 was compared to that of the Poly (3HB-*co*-7.1 mol% 3HHx) film.

**Figure 5:**
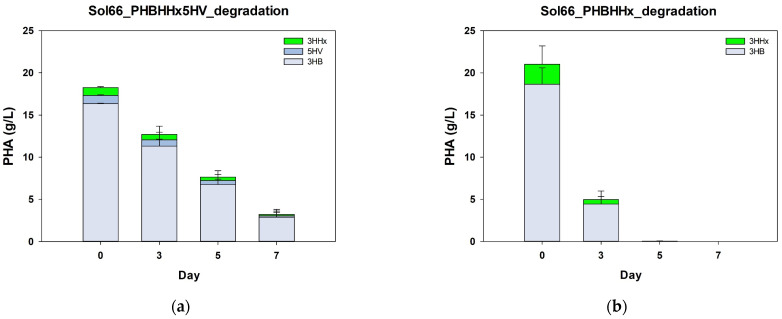
Time-dependent degradation rate of (**a**) poly(3HB-*co*-3.7 mol% 3HHx-*co*-5.3 mol% 5HV) and (**b**) poly(3HB-*co*-7.1 mol% 3HHx) films by *Microbulbifer* sp. Sol66. Statistical analysis was performed by applying ANOVA with the level of significance at 5%.

The Poly(3HB-*co*-3.6 mol% 3HHx-*co*-4.9 mol% 5HV) film exhibited a lower degradation rate than the Poly(3HB-*co*-7.1 mol% 3HHx) film. The Poly(3HB-*co*-7.1 mol% 3HHx) film degraded by 76.4% in just 3 days, whereas the Poly(3HB-*co*-3.6 mol% 3HHx-*co*-4.9 mol% 5HV) film degraded by only 30.4% (Figure 5). Additionally, while the PHBHHx film was completely degraded by the fifth day, the Poly(3HB-*co*-3.6 mol% 3HHx-*co*-4.9 mol% 5HV) film degraded by only 82.3% by the seventh day. Throughout the 7-day degradation period, the molar fraction of 5HV remained between 4 and 5%, and the molar fraction of 3HHx remained between 3 and 4%. This indicates that the degradation of PHA terpolymers by Sol66 did not specifically involve certain monomers.

**Figure 6:**
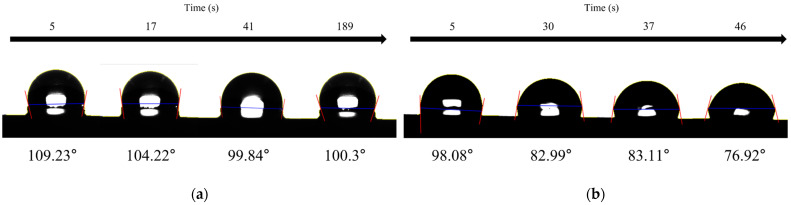
Analysis of changes in contact angle over time for extracted (**a**) poly(3HB-*co*-3.7 mol% 3HHx-*co*-5.3 mol% 5HV) and (**b**) poly(3HB-*co*-7.1 mol% 3HHx) films. Statistical analysis was performed by applying ANOVA with the level of significance at 5%.

The interaction of the two PHA films with liquids was analyzed using a contact angle meter. For poly(3HB-*co*-3HHx-*co*-5HV), the initial contact angle was 109.23°, which is higher than that of 98.08° for poly(3HB-*co*-3HHx) (Figure 6). This indicated that the poly(3HB-*co*-3HHx-*co*-5HV) film had a more hydrophobic surface. Furthermore, when observing the change in contact angle over time, Poly(3HB-*co*-3HHx-*co*-5HV) maintained a contact angle of approximately 100° for about 3 min. In contrast, the contact angle of the Poly(3HB-*co*-3HHx) film decreased to 76.92° after 46 s of measurement, and after 3 min, the water droplet was completely absorbed by the polymer. The PHA films showed differences in the contact angle depending on the presence of 5HV monomers. However, because the films may contain components derived from plant oils or microbial sources that can be extracted with chloroform and may influence the contact angle, it may be difficult to attribute these changes solely to the presence of 5HV.

## 4. Conclusions

Previous efforts to produce PHA copolymers or terpolymers through genetic modification of organisms aimed at synthesizing precursors from fructose or adding precursors alongside fructose have been limited by low production yields. Therefore, in this study, we aimed to explore the feasibility of mass-producing PHA containing 5HV by using vegetable oil together with DVL, a precursor of 5HV.

First, to efficiently incorporate 5HV into PHA, we tested the ability of three PHA synthases, known for their broad substrate specificity, to synthesize P(3HB-*co*-5HV). As a result, we developed a *Cupriavidus necator* PHB^−^^4^ strain harboring *phaC*_BP-M-CPF4_, which exhibited a very high ability to synthesize P(3HB-*co*-5HV). Additionally, by feeding bean oil, we found that while the addition of vegetable oil leads to further synthesis of 3HHx in the PHA, the overall PHA production increased by about 30% when DVL was used.

In a 5-L jar fermenter, the potential for large-scale production of poly(3HB-*co*-3HHx-*co*-5HV) through the addition of plant oil was confirmed. Under fed-batch fermentation, PHB^−^^4^/BP produced 48.63 ± 4.53 g/L of poly(3HB-*co*-3.7 mol% 3HHx-*co*-5.3 mol% 5HV) from 66.25 ± 2.95 g/L of DCW when 100 g/L of bean oil was additionally supplied. Furthermore, although the lag phase was long because the conditions were not thoroughly optimized, when 200 g/L of bean oil was supplied, 90.3 ± 0.9 g/L of DCW yielded 68.6 ± 7.8 g/L of poly(3HB-*co*-5.6 mol% 3HHx-*co*-2.7 mol% 5HV) at 144 h. The terpolymers were collected by continuous centrifugation and extracted with chloroform. By comparing the physical properties of the produced terpolymer and conventional PHBHHx, it was confirmed that, at lower mole fractions, the 3HHx monomer in PHA effectively increased the elongation at break, whereas the 5HV monomer effectively reduced the melting point.

The roles of 5HV and 3HHx in the produced PHA were elucidated through a comparison with conventional Poly(3HB-*co*-3HHx). Thermal and physical property analyses revealed that at low molar fractions, 3HHx effectively enhanced the elongation of PHA, while 5HV reduced its melting point. Additionally, PHA containing 5HV exhibited slower degradation by marine microorganisms compared to Poly(3HB-*co*-3HHx), with a higher contact angle, indicating improved water resistance.

Vegetable oil is an inexpensive feedstock known to produce significantly higher PHA yield and biomass in *Cupriavidus necator* fermentation compared to sugars. However, despite these advantages, the use of vegetable oil for PHA production has been primarily limited to P(3HB-*co*-3HHx), with few examples of its use for large-scale production of PHAs containing other monomers. In this study, we strategically utilized vegetable oil to enhance biomass production for the mass production of PHA containing 5HV. Furthermore, we propose using a combination of vegetable oil and precursors as an effective strategy for the large-scale production of PHAs with diverse monomers. The analysis of polymer properties suggests that incorporating 5HV can lower the melting point, regulate degradation rates in marine environments, and enhance water resistance. Given the demand for large quantities of samples in polymer applications, this oil-based fermentation strategy facilitates greater PHA production, offering expanded opportunities to investigate the properties of novel PHAs.

## Figures and Tables

**Table 1:** Production of co- or terpolymers with various monomers using *Cupriavidus necator* strains.

	C-Source	PHA Type	DCW (g/L)	PHA (g/L)	Content (%)	Ref.
Copolymers	fructose	P (3HB-*co*-2.1 mol% 3HP)	2.5	-	31	[18]
	fructose	P (3HB-*co*-37.7 mol% 3HHx)	1.42 ± 0.05	-	41.1 ± 3.0	[19]
	fructose	P (3HB-*co*-64.9 mol% 3HV)	1.5 ± 0.1	-	42.5 ± 3.8	[20]
	fructose + ε-CL	P (3HB-*co*-4HB)	7.5	-	-	[14]
	waste rapeseed oil + propanol	P (3HB-*co*-3HV)	14.7 ± 0.3	11.7 ± 0.7	80	[21]
		P (3HB-*co*-3HV) *	138	105	76	
	fructose + coconut oil	P (3HB-*co*-3HHx)	19	15	-	[22]
	palm kernel oil + butyrate	P (3HB-*co*-3HHx) *	153–175	113–138	73.3–78.6	[23]
Terpolymers	fructose + 4HVA + 5HVA	P (3HB-*co*-3HV-*co*-4HV-*co*-5HV)	8.7 ± 0.1	6.3 ± 0.1	72	[12]
	fructose + GVL	P (3HB-*co*-3HV-*co*-4HV)	8.2 ± 0.2	-	80 ± 2	[14]
	fructose	P (3HB-*co*-3HV-*co*-3H4MV-*co*-3H2MP)	1.67 ± 0.03	0.93 ± 0.03	55.9 ± 1.8	[16]
	tung oil	P (3HB-*co*-3HV-*co*-3HHx)	1.65	0.68	41.2	[24]
	fructose + bean oil + DVL	P (3HB-*co*-3HHx-*co*-5HV) *	66	49	73	this work
	fructose + bean oil + DVL	P (3HB-*co*-3HHx-*co*-5HV) *	90	69	77	this work

Abbreviation: ε-CL, ε-caprolactone; GVL, γ-valerolactone; 4HVA, 4-hydroxyvaleric acid; 5HVA, 5-hydroxyvaleric acid; DVL, δ-valerolactone; 3HB, 3-hydroxybutyrate; 3HV, 3-hydroxyvalerate; 3H4MV, 3-hydroxy-4-methylvalerate; 3H2MP, 3-hydroxy-2-methylpropionate; 3HP, 3-hydroxypropionate; 3HHx, 3-hydroxyhexanoate; 4HB, 4-hydroxybutyrate; 4HV, 4-hydroxyvalerate; 5HV, 5-hydroxyvalerate. PHA with the mark * was produced through fed-batch fermentation.

**Table 2:** Physical and mechanical properties of poly(3HB-*co*-3.6 mol% 3HHx-*co*-4.9 mol% 5HV) produced by *C. necator* PHB^−4^ harboring *phaC*_BP-M-CPF4_ and poly(3HB-*co*-7.1 mol% 3HHx).

PHA Composition			Properties					
3HB	3HHx	5HV	Tensile Strength (MPa)	Elongation at Break (%)	Young’s Modulus (MPa)	Mn (10^3^)	Mw (10^3^)	Dispersity
91.5	3.6	4.9	9.03 ± 0.18	16.64 ± 1.36	112.14 ± 4.65	60 ± 10	306 ± 19	5.2 ± 0.5
92.9	7.1	-	6.81 ± 0.42	176.6 ± 18.24	81.71 ± 5.25	53 ± 3	354 ± 11	6.7 ± 0.2

**Table 3:** Thermal properties of poly(3HB-*co*-3.6 mol% 3HHx-*co*-4.9 mol% 5HV) produced by *C. necator* PHB^−4^ harboring *phaC*_BP-M-CPF4_ and poly(3HB-*co*-7.1 mol% 3HHx).

	Tg (°C)	Tc (°C)	Tm (°C)	ΔH (mJ/mg)
Poly(3HB-*co*-3HHx-*co*-5HV)	n.a.	n.a.	151.5 ± 0.1	16.6 ± 0.3
Poly(3HB-*co*-3HHx)	2.6 ± 0.2	48.3 ± 0.3	175.5 ± 0.0	19.2 ± 0.2

Abbreviations: Tg, glass transition temperature; Tc, crystallization temperature, Tm, melting temperature.

## Data Availability

Data will be available on request.

## Supplementary Materials

The following supporting information can be downloaded at: https://www.mdpi.com/article/10.3390/polym16192773/s1, Figure S1: Fed-batch Poly(3HB-*co*-3HHx-*co*-5HV) production by *Cupriavidus necator* PHB-4 harboring *phaC*_BP-M-CPF4_ in a 5-L jar fermenter. The initial culture conditions included 1% fructose, 0.5% bean oil, and 0.1% NH4NO3. From 10 h to 20 h of culture, 200 g/L of bean oil was supplied, and 5 g/L of DVL was added after 48 h. The changes in (A) DCW (Dry Cell Weight) and PHA, as well as (B) the molar fractions of 3HHx and 5HV were monitored over the cultivation period.
