# Supplementary material for: Strategic Use of Vegetable Oil for Mass Production of 5-Hydroxyvalerate-Containing Polyhydroxyalkanoate from δ-Valerolactone by Engineered Cupriavidus necator
Source: Polymers (Basel). 2024 Sep 30;16(19):2773. doi: 10.3390/polym16192773 (PMC11478691; doi:10.3390/polym16192773)
Supplement: Supplementary file 1 [file polymers-16-02773-s001.zip › polymers-3193179-supplementary.pdf]

Supplementary Figure S1.

(A)

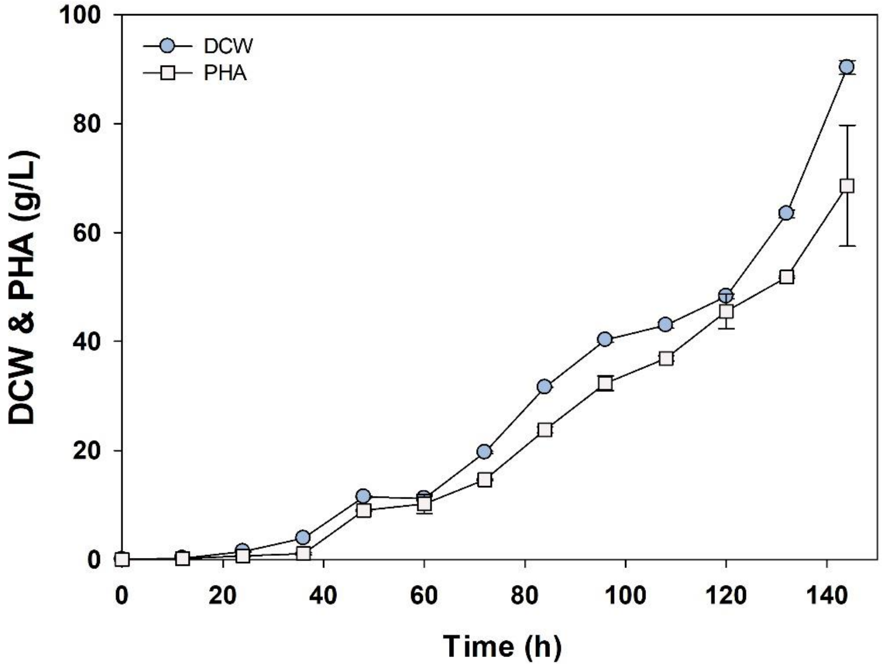

(B)

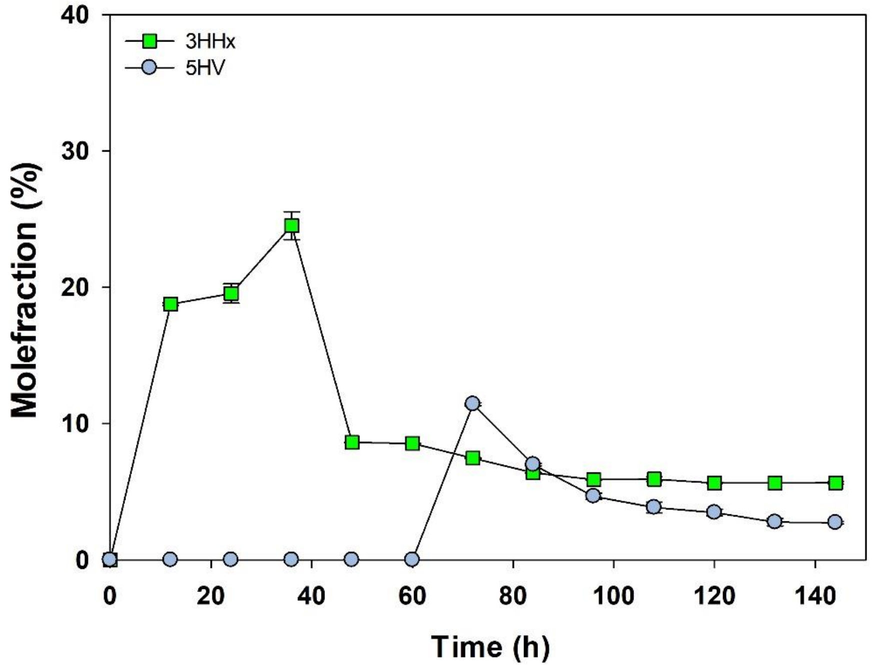

Figure S1. Fed-batch Poly(3HB-*co*-3HHx-*co*-5HV) production by *Cupriavidus necator* PHB^{-4} harboring *phaC*$_{BP-M-CPF4}$ in a 5-L jar fermenter. The initial culture conditions included 1% fructose, 0.5% bean oil, and 0.1% NH_4NO_3. From 10 h to 20 h of culture, 200 g/L of bean oil was supplied, and 5 g/L of DVL was added after 48 h. The changes in (A) DCW (Dry Cell Weight) and PHA, as well as (B) the molar fractions of 3HHx and 5HV were monitored over the cultivation period.
